# Aortic obstruction: anatomy and echocardiography

**DOI:** 10.1186/1476-7120-4-36

**Published:** 2006-09-29

**Authors:** Nilda Espinola-Zavaleta, Luis Muñoz-Castellanos, Magdalena Kuri-Nivon, Candace Keirns

**Affiliations:** 1 Echocardiography in Out Patient's Clinic, Instituto Nacional de Cardiología "Ignacio Chávez" Juan Badiano N° 1, Colonia Sección XVI, Tlalpan, Mexico City, Mexico; 2 Department of Embryology, Instituto Nacional de Cardiología "Ignacio Chávez". Juan Badiano N° 1, Colonia Sección XVI, Tlalpan, Mexico City, Mexico; 3 Morphology Department, Escuela Superior de Medicina-IPN. Diaz-Mirón y Plan de San Luis, Colonia Casco de Santo Tomás, Tacuba, Mexico City, Mexico

## Abstract

Echocardiography is a valuable non-invasive technique for identifying the site and type of aortic obstruction. Knowledge of the morphological details of each type of obstruction is the basis for correct interpretation of the diagnostic images and clinical decisions. This study was undertaken to correlate the echocardiographic images with anatomic specimens of equivalent valvular and supravalvular aortic obstruction. Specimens were part of the collection of the Department of Embryology. Fifty six patients were studied, and forty specimens with aortic obstruction were analyzed.

**Echocardiographic characteristics**: Thirty one (55.3%) patients were women and twenty five (44.7%) men. Valvular aortic obstruction was found in Thirty six patients (64.3 %) and supravalvular aortic obstruction in twenty (35.7%). **Anatomic characteristics**: Of the forty specimens examined, twenty one (52.5%) had valvular aortic obstruction and nineteen (47.5%) supravalvular aortic obstruction.

The anatomoechocardiographic correlation clearly showed that the anatomic findings of the specimen hearts and aortas corresponded to echocardiographic images of valvular and supravalvular aortic obstruction and provided solid corroboration of echocardiographic diagnoses.

## Background

Obstructions of the aortic valve are determined by the dimensions of the fibrous ring, the number and morphological alterations of the semilunar leaflets that may be bicuspid, tricuspid, or quadricuspid, dysplastic thickening of the valve and fusion of the commissures [1,3]. Forms of supravalvular obstructions include hourglass, diffuse and diaphragmatic. They may involve obstructions of the ascending aorta such as tubular hypoplasia and hypoplasia of the aortic arch as well as coarctation of the descending aorta [4,5].

All types of obstruction cause left ventricular hypertrophy, which creates an imbalance between the supply and demand for oxygen, leading to myocardial ischemia, heart failure and death if a precise diagnosis is not established and timely treatment instituted.

Echocardiography is a noninvasive technique of great value in identifying the site and type of aortic obstruction, in evaluating associated anomalies and their hemodynamic significance [6-9] and in planning surgical correction [10]. Knowledge of the morphological details of each obstructive type constitutes the basis for correctly interpreting the diagnostic images and making clinical decisions. The aim of this study was to correlate the echocardiographic images of patients with different types of aortic obstruction with those of equivalent anatomic specimens from the collection of specimens in the Department of Embryology of the Instituto Nacional de Cardiología Ignacio Chávez.

## Methods

Between May 2003 and May 2005 fifty six patients with obstruction of the aortic valve, ascending aorta, aortic arch and descending aorta were studied. Aortic valve obstruction was considered to be an obstruction produced by alterations in the number and morphology of the aortic leaflets, in the dimensions of the fibrous ring, thickening of the leaflets and fusion of commissures. Supravalvular aortic obstruction was defined as narrowing of the lumen immediately above the valve. This last could be localized or diffuse and involve the ascending aorta or portions of the aortic arch. Coarctation was defined as a narrowing of the descending aorta.

Transthoracic and/or transesophageal echocardiography was performed on all patients using Hewlett Packard Sonos 5500 equipment with an S3 transthoracic probe and a multiplane transesophageal probe. In some cases, particularly for cases of bicuspid aorta, sections were acquired for three-dimensional off-line reconstruction.

Aortic valve obstructions were visualized in short axis sections at the level of the great arteries, while supravalvular obstructions of the ascending aorta were assessed in parasternal long axis images. Suprasternal sections were used to view obstructions of the aortic arch and descending aorta.

In cases of poor visualization, especially in aortic valve and ascending aorta, transesophageal technique was utilized at 30–50° and 120–130°.

Off-line three-dimensional reconstruction of transesophageal images was performed by taking sections every 2° from 0° to 180° with electrocardiographic and respiratory synchronization. The data from sections in the region of interest were stored in optic discs and later transferred to a computer containing software for three-dimensional off-line reconstruction (Echo-scan, version 3.1, TomTec Gmb H, Munich, Germany), as described before [11].

In addition, the morphological findings of 40 heart specimens with different types of aortic obstruction from the Institute's Department of Embryology were analyzed. Each specimen was described following the guidelines of the segmental sequential system used in the diagnosis of congenital heart disease [12]. These were atrial situs, atrioventricular and ventriculoarterial connections, morphology of atrioventricular valves, obstructions of the aortic valve, ascending aorta, aortic arch and descending aorta, as well as morphological characteristics of the ventricles, including the ventricular septum. The morphologic features of equivalent heart specimens were compared with the corresponding patient's echocardiographic images to provide the anatomic bases of the latter.

## Results

Echocardiographic and anatomic characteristics of the two groups are shown in Tables 1 and 2.

**Table 1:** Echocardiographic findings

**Type**	**Pathology**	**No. of Patients**	**Percent**
**Valvular **36 (64.3 %)	Bicuspid aorta	31	55.3
	Tricuspid aorta	2	3.6
	Quadricuspid aorta	2	3.6
	Bicuspid aorta and coarctation	1	1.8
**Supravalvular **20 (35.7%)	Fibrous ring (hourglass)	1	1.8
	Tubular hypoplasia of the ascending aorta	1	1.8
	Hypoplasia of the aortic arch	1	1.8
	Coarctation of the aorta	17	30.3
**Total**		56	100

**Table 2:** Anatomopathological findings

**Type**	**Pathology**	**No. of patients**	**Percent**
**Valvular **21 (52.5 %)	Bicuspid aorta	20	50
	Quadricuspid aorta	1	2.5
**Supravalvular **19 (47.5 %)	Fibrous ring (hourglass)	3	7.5
	Tubular hypoplasia of the ascending aorta	3	7.5
	Hypoplasia of the aortic arch	5	12.5
	Coarctation of the aorta	8	20
**Total**		40	100

### Echocardiographic characteristics

Thirty one (55.3%) patients were women and twenty five (44.7%) men. The average was 31.50 years (range 18 to 70). Thirty-six (64.3%) had aortic valve obstruction. The aortic valve was bicuspid in thirty one, tricuspid in two, quadricuspid in two and bicuspid associated with coarctation of the aorta in one. In twenty cases (35.7%) the aortic obstruction was supravalvular. One patient with Williams's syndrome had an hourglass fibrous ring, one tubular aortic hypoplasia, and one hypoplasia of the aortic arch and seventeen coarctation of the aorta (Table 1).

### Anatomic characteristics

Of the forty specimen hearts studied, twenty-one (52.5%) had aortic valve obstruction. Of these twenty were bicuspid and one quadricuspid. Nineteen (47.5%) had supravalvular obstruction with hourglass fibrous rings in three, tubular hypoplasia of the ascending aorta in three, hypoplasia of the aortic arch in five and coarctation of the aorta in eight (Table 2).

### Anatomoechocardiographic correlation

A comparison of echocardiographic images with aspects of specimen hearts and aorta with equivalent obstructive subtypes clearly showed findings that corresponded closely.

**Figure 1:**
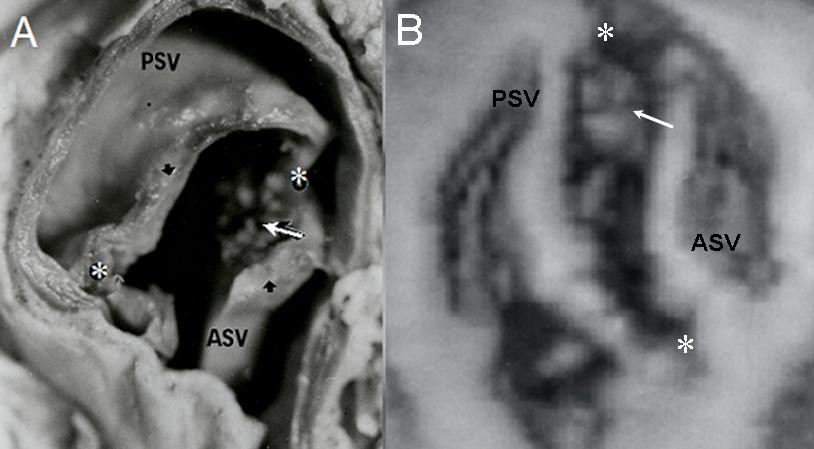
Bicuspid aorta. Anatomic specimen (A) and 3-D reconstruction of echocardiographic images (B) show anterosuperior relationship of aortic leaflets with thickened edges (black arrowheads in A), nodules on the internal aspect (arrows) and lateralized commissures (asterisks). ASV: Anterior leaflet; PSV: Posterior leaflet.

Bicuspid aortic obstruction demonstrated thickened aortic leaflets with dysplastic nodulations on both faces with calcified area. In thirteen specimens the aortic valves had two leaflets. The position of the commissures in the anatomic specimens was lateral (right to left) in eight with anteroposterior leaflets, while in five specimens the commissures were anteroposterior with lateral leaflets. In one case the bicuspid aortic valve was associated with a subvalvular fibrous crest and subvalvular thickening. In seven specimens 3 commissures were identified, one of which presented variable degrees of involution with fusion of the adjacent aortic leaflets, which created an acquired bicuspid valve. The anatomic findings correlated with the three-dimensional reconstruction of the echocardiographic image (Figure 1).

**Figure 2:**
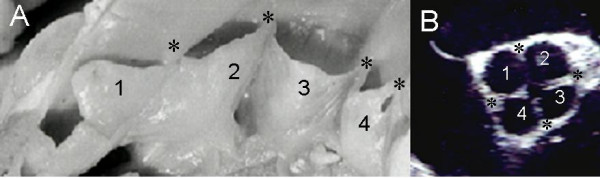
Quadricuspid aorta. Photograph (A) and transesophageal image at 50° (B) of aortic valves with four leaflets (1,2,3, and 4) and four commissures (asterisks).

The anatomic specimen of a quadricuspid aorta showed that the four leaflets and four commissures were thickened. These characteristics produced the echocardiographic image of a cross (Figure 2).

**Figure 3:**
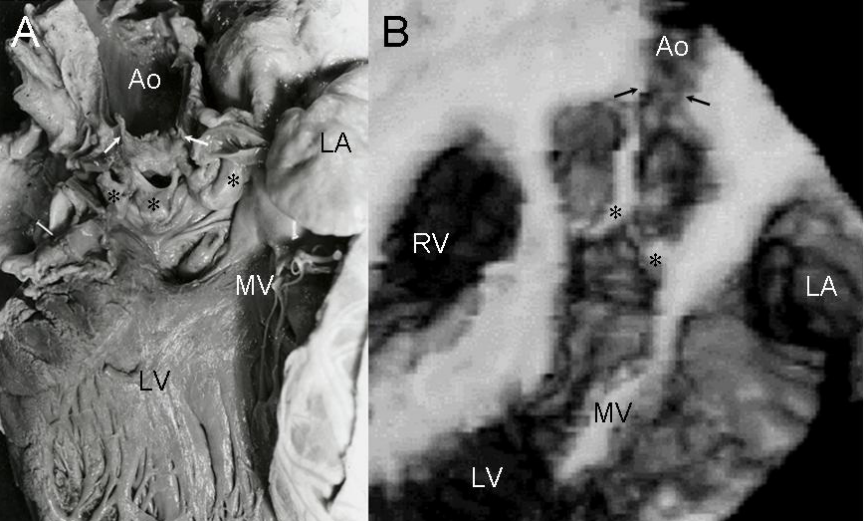
Supravalvular aortic stenosis. Anatomic specimen (A) and 3-D reconstruction of transesophageal image at 120° shows obstruction above the aortic leaflets (asterisks). Abbreviations as before.

Fibrous supravalvular obstruction showed the hourglass configuration with narrowing evident above the aortic valve in the anatomic specimen and echocardiographic image (Figure 3).

**Figure 4:**
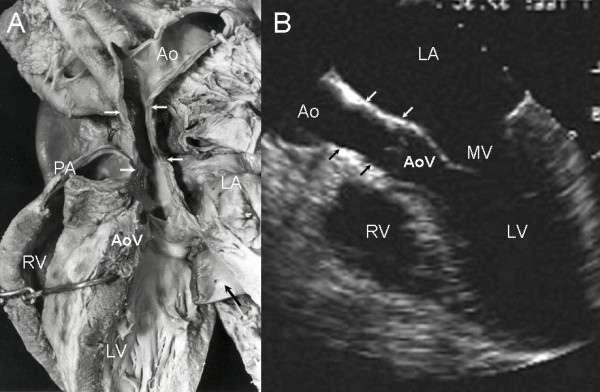
Tubular hypoplasia of the ascending aorta. Anatomic specimen (A) and four-chamber transesophageal image (B) demonstrate narrowing of the ascending aorta (white arrows in A). The black arrow in (A) points to a biological prosthesis in mitral position. AoV-Aortic valve, PA-Pulmonary artery. Other abbreviations as before.

**Figure 5:**
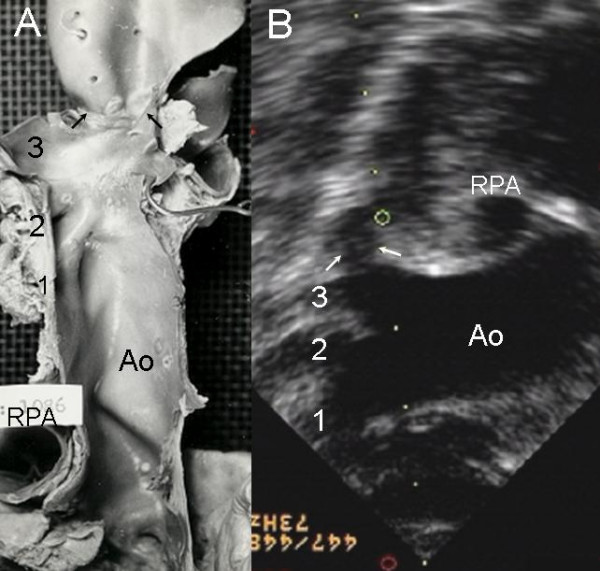
Coarctation of the aorta. (A) Aorta with narrow lumen (black arrows). (B) Suprasternal echocardiographic image reveals the site of the obstruction distal to the left subclavian artery (white arrows). RPA: Right pulmonary artery; 1-Brachiocephalic artery; 2-Left carotid artery; 3-Left subclavian artery.

**Figure 6:**
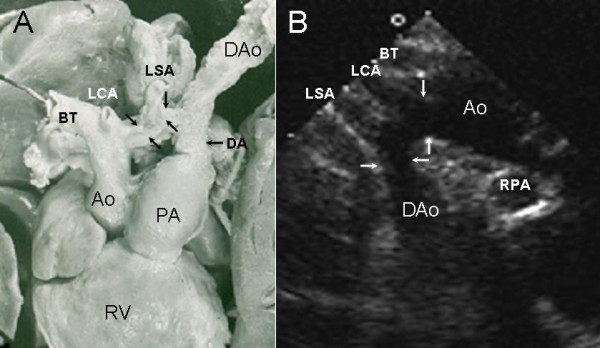
Hypoplasia of the horizontal portion of the aortic arch. Anatomic specimen (A) and suprasternal echocardiogram (B) show stenosis (hypoplasia) between the left carotid and subclavian arteries (black arrows in A, white arrows in B). LSA: Left subclavian artery; LCA-Left carotid artery, BT: Brachiocephalic artery; DA: Ductus arteriosus, DAo: Descending aorta. Other abbreviations as before.

Tubular hypoplasia of the aorta manifested as a diffuse narrowing above the aortic leaflets and correlated well with echocardiographic findings (Figure 4). Hypoplasia of the aortic arch demonstrated narrowing in the horizontal portion between the left carotid artery and subclavian arteries (Figure 5). Coarctation of the aorta in a specimen from an adult corresponded closely to the echocardiographic finding (Figure 6).

## Discussion and conclusion

Obstruction of the aortic valve occurs frequently [1]. It can be the result of thickening and calcification of a congenitally bicuspid valve or a bicuspid acquired valve originated by the fusion of two leaflets or by thickening of a tricuspid or quadricuspid valve, although the last is more often associated with aortic regurgitation and rarely obstructive [7,13,14].

Supravalvular aortic obstruction occurs above dilated aortic sinuses that protrude laterally. The leaflets are often mildly thickened; the coronary arteries are thickened and dilated. The most common form is hourglass shaped with dilatation of the distal aorta. Diafragmatic and diffuse tubular variants also exist. The wall of the ascending aorta is thickened with disorganization of the medial and intimal layers. These alterations can also be found in the abdominal arteries. In 1961 Williams reported the association of supravalvular aortic stenosis with elfin facies and mental retardation now known as Williams's syndrome [5]. Tubular hypoplasia of the aorta is characterized by diffuse narrowing distal to the valve. Hypoplasia of the aortic arch involves narrowing in its horizontal portion between the left carotid and subclavian arteries [4,15,16]. Mature onset coarctation of the aorta is located distal to the origin of the left subclavian artery [17,18].

Indications for surgical therapy are based on clinical and hemodynamic parameters, and these data can now be obtained by two-dimensional and Doppler echocardiography rather than cardiac catheterization. The success rate of the outcome after surgery depends on the nature of the morphology and the adequacy of surgical correction [10].

Echocardiography, especially three-dimensional reconstruction, makes it possible to obtain a data set that can be scanned to determine the smallest luminal area and the degree of obstruction, as well as its length providing more realistic information with depth perception. The extent and severity of the aortic lesions can be adequately characterized and may provide data that allow a more specific surgical approach. Various authors have demonstrated the value of different echocardiographic techniques in the diagnosis of aortic obstructions [9,15].

The anatomo-echocardiographic correlation can be done, because all hearts with a type of congenital heart disease have the same basic morphology, which permits this comparison; the heart specimens were selected with similar anatomic features which match with the echocardiographic images of the patients, so the figures show same pathology in different patients. We believe that the value of this comparison lies in its contribution to a precise diagnosis, leading to early and appropriate treatment of patients with valvular and supravalvular aortic obstruction.

## Competing interests

The author(s) declare that they have no competing interests.

## Authors' contributions

We would like to report specific contribution of each author of the manuscript: NEZ performed and interpreted the echocardiographic studies and participated in the design of the study and in the anatomo-echocardiographic correlation. LMC made the dissections of the hearts with aortic obstructions and participated in the design of the study and in the anatomo-echocardiographic correlation. MKN made the photographs of heart specimens and helped to draft the manuscript. CK helped to draft the manuscript and made the translation from spanish into English.

All authors read and approved the final manuscript.
